# Rapid adaptation and extinction across climates in synchronized outdoor evolution experiments of *Arabidopsis thaliana*

**DOI:** 10.1101/2025.05.28.654549

**Published:** 2025-05-28

**Authors:** Xing Wu, Tatiana Bellagio, Yunru Peng, Lucas Czech, Meixi Lin, Patricia Lang, Ruth Epstein, Mohamed Abdelaziz, Jake Alexander, Mireille Caton-Darby, Carlos Alonso-Blanco, Heidi Lie Andersen, Modesto Berbel, Joy Bergelson, Liana Burghardt, Carolin Delker, Panayiotis G. Dimitrakopoulos, Kathleen Donohue, Walter Durka, Gema Escribano-Avila, Steven J. Franks, Felix B. Fritschi, Alexandros Galanidis, Alfredo Garcia-Fernández, Ana García-Muñoz, Elena Hamann, Martijn Herber, Allison Hutt, José M. Iriondo, Thomas E. Juenger, Stephen Keller, Karin Koehl, Arthur Korte, Pamela Korte, Alexander Kuschera, Carlos Lara-Romero, Laura Leventhal, Daniel Maag, Arnald Marcer, Martí March-Salas, Juliette de Meaux, Belén Méndez-Vigo, Javier Morente-López, Timothy C. Morton, Zuzana Münzbergova, Anne Muola, Meelis Pärtel, F. Xavier Picó, Brandie Quarles-Chidyagwai, Marcel Quint, Niklas Reichelt, Agnieszka Rudak, Johanna Schmitt, Merav Seifan, Basten L. Snoek, Remco Stam, John R. Stinchcombe, Marc Stift, Mark A. Taylor, Peter Tiffin, Irène Till-Bottraud, Anna Traveset, Jean-Gabriel Valay, Martijn van Zanten, Vigdis Vandvik, Cyrille Violle, Maciej Wódkiewicz, Detlef Weigel, Oliver Bossdorf, Robert Colautti, François Vasseur, J.F. Scheepens, Moises Exposito-Alonso

**Affiliations:** 1 Department of Integrative Biology, University of California Berkeley, Berkeley 94720, CA, USA.; 2 Howard Hughes Medical Institute, University of California Berkeley, Berkeley 94720, CA, USA.; 3 Department of Plant Biology, Carnegie Institution for Science, Stanford 94305, CA, USA.; 4 Department of Biology, Stanford University, Stanford 94305, CA, USA.; 5 CEFE, Univ Montpellier, CNRS, EPHE, IRD, Montpellier, France.; 6 Faculty of Biological Sciences, Goethe University Frankfurt, Max-von-Laue-Str. 13, 60438 Frankfurt, Germany.; 7 Department of Global Ecology, Carnegie Institution for Science, Stanford 94305, CA, USA.

## Abstract

Climate change is threatening species with extinction, and rapid evolutionary adaptation may be their only option for population rescue over short ecological timescales. However, direct observations of rapid genetic adaptation and population dynamics across climates are rare across species. To fill this gap, we conducted a replicated, globally synchronized evolution experiment with the plant *Arabidopsis thaliana* for 5 years in over 30 outdoor experimental gardens with distinct climates across Europe, the Levant, and North America. We performed whole-genome sequencing on ~70,000 surviving reproductive individuals and directly observed rapid and repeatable adaptation across climates. Allele frequency changes over time were parallel in experimental evolution replicates within the same climates, while they diverged across contrasting climates—with some allele frequency shifts best explained by strong selection between −46% to +60%. Screening the genome for signals of rapid climate adaptation identified a polygenic architecture with both known and novel adaptive genetic variants connected to important ecological phenotypes including environmental stress responses, *CAM5* and *HEAT SHOCK FACTORs,* and germination and spring flowering timing*, CYTOCHROME P450s* and *TSF*. We found evolutionary adaptation trends were often predictable, but variable across environments. In warm climates, high evolutionary predictability was associated with population survival up to 5 years, while erratic trends were an early warning for population extinction. Together, these results show rapid climate adaptation may be possible, but understanding its limits across species will be key for biodiversity forecasting.

Rapid evolutionary adaptation at ecological time scales in the wild has been documented across eukaryotes, from field mustard (1), barley (2), and Darwin’s finches (3), to fruit flies (4), stick insects (5), and sticklebacks (6). Despite evidence of rapid adaptation in natural environments, it is still unknown the extent to which rapid adaptation can rescue vulnerable populations from climate-change-driven population and species extinctions (7, 8), but we still do not fully understand the tempo, dynamics, and predictability of rapid evolutionary adaptation in complex climates and organisms. The gold standard to experimentally study the dynamics of evolution has been represented by microbial long-term laboratory experiments combined with genome re-sequencing (9, 10)—where evolutionary adaptation typically occurs via *de novo* mutations over thousands of generations—and by animal and plant field experiments as in fruit fly orchards (11, 12) or domesticated plant trials (2)—where generation times are longer, population sizes are smaller, and rapid adaptation often occurs via selection on standing pre-existing genetic variation (13). However, to address major unanswered questions on rapid evolution to climate and population responses we are still lacking large-scale animal or plant experimental evolution replicated across continents over years.

Common garden and reciprocal transplant experiments, pioneered with plants (14), are powerful methods for comparing fitness across multiple genotypes of a species from different locales in the same environment. These approaches have been key tools to reveal pervasive within-species standing variation and past climate adaptation across macro-organisms (15–17). However, knowledge about within-species variation only teaches us about the species potential for adaptation, but is not a direct observation of adaptation dynamics or population rescue occurring in time. Here, we study the process of rapid evolution over 5 years by melding common garden experiments in multiple climates and genome re-sequencing in the annual plant *Arabidopsis thaliana*. We find evidence of rapid evolutionary responses to climate, identify novel genes, and predict short-term evolutionary trends. However, evolutionary adaptation was not possible across all environments, highlighting the importance of understanding not only the potential but also the limits of evolution under the exceptional pressures raised by climate change.

## A synchronized evolution experiment in *Arabidopsis thaliana* across climates

**Fig. 1.**
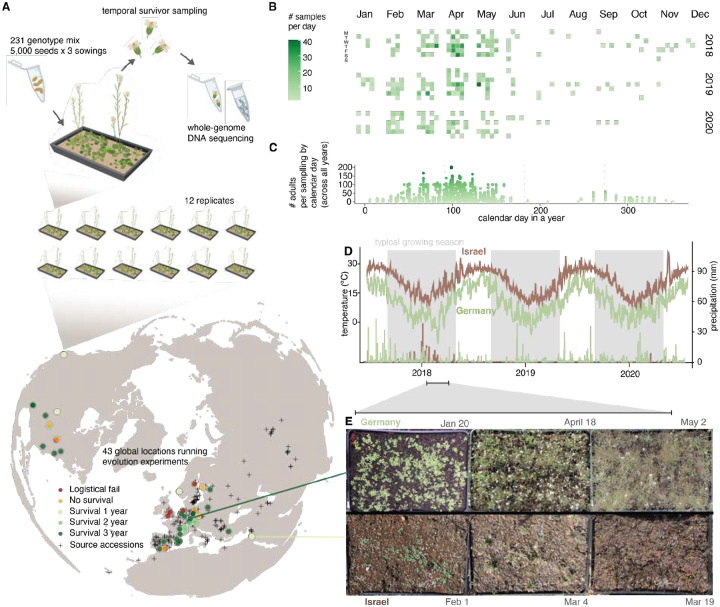
GrENE-net’s globally-distributed evolution experiment of *A. thaliana*. (**A**) GrENE-net experimental design with 231 *A. thaliana* accessions mixed in tubes of ~5,000 seeds. Each experimental tray was sown with three tubes and seeds were spread every two weeks throughout fall 2017 to ensure establishment. Each site started 12 trays as independent experimental replicates. The map shows 43 gardens (sites) where participants started the experiment; colors indicate experiment outcomes, with 30 sites successfully completing at least one generation and producing genomic data. (**B**) Calendar of time-series collections of flower tissues used for genomic sequencing for the first three years. (**C**) Density of samples collected along the calendar year, combining data from all three years. (**D**) Daily temperatures curves and precipitation bars over the first three years of the experiment in two example locations: humid continental (Würzburg, Germany, site #46, green) and arid desert (Sde Boker in Negev desert, Israel, site #26, brown). (**E**) Example photographs of the experimental populations in Germany and Israel during spring of the first growing season.

To understand the genetic basis of rapid evolution across a wide range of climates, we conducted a multi-year and multi-location evolution experiment in outdoor gardens, using the hermaphroditic highly-selfing annual plant *Arabidopsis thaliana* as a model species. We established the GrENE-net consortium (www.GrENE-net.org) to implement a simplified and standardized protocol for evolution experiments (see protocol, Text S1). Experiments were coordinated across 43 locations across Europe, the Levant, and US (Fig. 1, Fig. S1, Table S1), and began in the fall of 2017. Experimental sites spanned contrasting climates—from urban European environments to the likely edge of the species’ niche, the Negev desert. At each location, 12 independent replicate trays of plants were established and maintained for up to five years (*A. thaliana* typically undergoes one generation per year with a spring flowering, or two generations in some climates with spring and fall flowering). Experimental trays were filled with homogenized soil, tagged with temperature and humidity sensors, placed outdoors to grow with minimal human intervention (i.e. no watering, fertilization, or shelter), and sown with ~15,000 seeds of the same founder genotypes: 231 *A. thaliana* accessions. These accessions were selected to represent the entire native geographic range of *A. thaliana* and were mixed at roughly equal proportions and validated by whole-genome sequencing (Fig. 1A, Table S2, see sequence validation in Text S4, S5, Dataset S4, S5, Fig. S12, S13). The population in each tray was allowed to reproduce naturally, allowing for the study of demography and evolution across generations and climates. In predominantly self-fertilizing populations, adaptation is expected to advance through the differential fitness of inbred lineages, whereas any outcrossing that does occur generates recombinant genotypes on which selection can act at a finer genomic scale. We note that we detected signs of outcrossing in ~6–16% of samples (see Text S10, Fig. S14) in line with observations from natural populations (18). Out of 43 initial sites, seven sites failed due to experimental logistics, and six sites established populations that all died off within a few months, possibly due to extreme climatic conditions. Plants from the remaining 30 sites successfully reproduced for at least one generation and yielded high-quality genomic and demographic data (Fig. S5).

Here, we present genomic data from the first three years of GrENE-net (2017–2020) along with complete census and environmental data (2017–2022) (Phase I data release, www.GrENE-net.org/data and Supplemental Materials). This includes daily climate data (Dataset S2), biweekly per-tray photographs from the growing seasons (Dataset S3), 1,141 demographic measurements (Fig. S5, Dataset S1, Table S3) and 74,491 tissue samples of reproductive plants collected and sequenced in 2,415 pools (Fig. S4, Fig. S2, Table S5) (19). Each genomic DNA library was generated from 1 to ~200 pooled flowers collected from a single tray of surviving plants at standardized sampling times, providing a snapshot of the genetic makeup of the reproductive population (Fig. 1A–C, Text S1, S2). For each sample, we reconstructed allele frequencies for ca. 3.2 million single nucleotide polymorphisms (SNPs) with estimated ~0.7% error rate. To achieve this, we combined allele count information from ~10X coverage of pool-sequencing per experimental population sample and sampling error reduction using linkage in the 231 founder accessions from high-coverage sequencing (Text S5, Fig. S11, S12, S13, Dataset S6, S7), following established methods from the evolve & resequence literature (20, 21). The composition of 231 founder accessions in the starting seed mix was also reconstructed by high coverage sequencing of the seed mix, confirming a very even representation of ≈0.05% per accession (i.e. 1/231) with 0.1% error rate (Text S5, Fig. S12). By merging data from multiple samplings of the same tray and growing season, we generated genome-wide allele frequencies and accession relative frequencies of 738 replicated populations across 30 locations, spanning up to 12 replicate populations per location and up to three sequenced years (i.e. equivalent to ≈3–6 generations in addition to starting generation). Combining these paired environmental and demographic metadata with evolutionary trajectories, we then studied the patterns and genomic architecture of rapid evolution across climates.

## Evolution is rapid across climates in GrENE-net

To directly study rapid adaptation from standing genetic variation, we analyzed genome-wide allele frequency changes and population differentiation across all generations and experimental gardens. We reason that allele frequency trends that are significantly synchronized (increasing or decreasing) across independent population replicates within one garden (i.e. repeatable adaptation) or different gardens of similar climate (i.e. parallel adaptation) must reflect the action of natural selection.

**Fig. 2.**
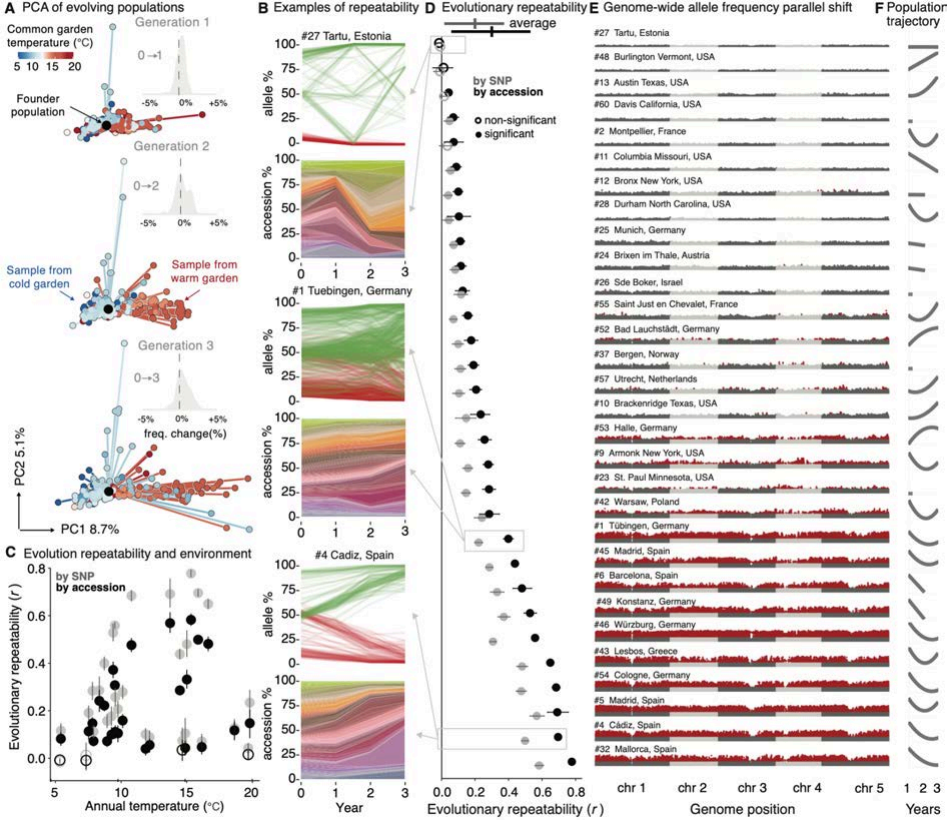
Genomic evolution in GrENE-net is rapid and parallel (**A**) Principal Component Analysis (PCA) of allele frequencies and samples over three generations with up to 12 replicates per location (n=738). The genome-sequenced founder population, common to all experiments, was projected into the PCA space (black). Insets show the distribution of genome-wide allele frequency changes between generations. (**B**) Example of three sites with low, intermediate, and high evolutionary repeatability displayed at the allele and accession level. At the allele level, the 100 fastest increasing or decreasing allele frequencies over time are plotted for illustration. At the accession level, all 231 accessions are displayed using a Muller plot with accessions sorted based on the temperature of origin from colder (green-yellow) to warmer (purple-blue). (**C-D**) Evolutionary repeatability measured at the allele or accession level as an average correlation of change in frequency from the founder frequency to first generation is displayed against the (**C**) garden annual temperature or (**D**) as a vertical rank. (**E**) Manhattan plots of Genome-Wide Likelihood Ratio Tests (LRTs) of alleles changing in frequency across 12 replicates within a site in the first generation (red indicates alleles with significant natural selection under Bonferroni correction). (**F**) Population trajectories of each location estimated across all years and replicates displaying the fitting of a polynomial regression (for expanded visualization Fig. S8).

We first measured the degree of population differentiation by comparing shifts in allele frequencies between starting founder and evolved populations across time and space using *F*_ST_ (22). We observed that the median *F*_*ST*_ across all experimental gardens with respect to the founder population increased with each generation, indicating the gradual differentiation from the founder population over time (across all samples: *F*_*ST* y1 median [IQR]_ = 0.002 [0.001–0.006], *n* = 319, *F*_*ST* y2 median [IQR]_ = 0.017 [0.010–0.036], *n* = 217, *F*_*ST* y3 median [IQR]_ = 0.024 [0.010–0.055], *n* = 182, Fig. S19A). In addition, *F*_*ST*_ divergence was significantly larger between-gardens compared to within-gardens (Mann-Whitney U Test *P* = 2×10^−90^, *n* = 50,403 pairwise combinations, Fig. S19B). To better understand population divergence across environments, we then decomposed allele frequency changes of evolved populations across all gardens using a principal component analysis and diffusion maps (Fig. 2A, Fig. S20 S21). The major axes of allele frequency change separate evolved populations according to the climate of the experimental gardens they were planted in, where experiments in similar climates led to similar evolutionary trajectories and vice versa (Pearson’s correlation PC1-annual mean temperature [BIO1], *r* = 0.436, *P* = 2.348×10^−10^, *n* = 193; Pearson’s correlation PC2-precipitation of wettest month [BIO13], *r* = 0.248, *P* = 4.918×10^−4^, *n* = 193).

To test whether the observed magnitude of evolution was significantly larger than expected from neutral genetic drift, we compared the observed variance of allele frequency changes (*Var(*Δ*p)=(p*_*1*_−*p*_*0*_*)*^*2*^) with neutral evolution expectations from theory and simulations. Our rationale is that genetic drift naturally creates shifts in allele frequencies from just stochastic sampling (measured genome-wide as *Var(*Δ*p)*). Then, if we find larger observed shifts than expectations of shifts from several stochastic demographic processes, we will need to invoke other evolutionary forces. First, comparing observed frequency variance in experimental populations with the classic Wright-Fisher population expectation (*Var*_*WF*_=*Var(*Δ*p)*_*WF*_=*p*_*0*_*(1-p*_*0*_*)/2N*), we found on average 3-fold larger shifts across samples of different population sizes and starting allele frequency classes (*Var*_*observed*_*/Var*_*WF*_ = 2.99 [CI_95%_=2.94–3.04], Mann-Whitney U Test *P*<2×10^−16^; assuming *N* as the sample size sequenced, which yields a conservative test, Fig. S16, S17, Text S7). Larger allele frequency deviations would be expected if WF assumptions are violated, such as due to lack of complete outcrossing or equal reproduction contributions common in naturally evolving *A. thaliana* populations. We then conducted another set of non-WF neutral expectation, by simulating random accession sorting (i.e. no outcrossing) and unequal reproduction (i.e. uniform or Poisson distributed seed set) (see Text S7). We still found significantly larger observed frequency changes than expected under non-WF sorting dynamics, especially in experimental replicates of larger population sizes (Fig. S17), with increasing deviations of observed evolution from neutral expectations over generations (t_0_→t_1_:*Var*_*observed*_*/Var*_*neutral-sorting*_ = 4.155 [95% CI: 2.576–5.734], t_0_→t_2_:*Var*_*observed*_*/Var*_*neutral-sorting*_ = 9.131 [95% CI: 7.167–11.095], t_0_→t_3_:*Var*_*observed*_*/Var*_*neutral-sorting*_ = 8.398 [95% CI: 6.584–10.211], Fig. 2A, Fig. S15, S16, S17, Text S7, S8).

Having established that allele frequency changes significantly depart from several neutral stochastic expectations, we hypothesize that environment-driven natural selection may have created larger, deterministic allele trajectories. Such natural selection should create repeatable allele frequency shifts in multiple replicates in the same garden (i.e. correlation of [*p*_*1*_*-p*_*0*_] between replicate *i* and *j*, see Text S9). We found significant evolutionary repeatability in 24 out of 30 gardens, as shown by high rank correlations of genome-wide allele frequency trends (Fig. 2B–D, Fig. S24, S25, S25, S27, S28, mean[sd] *r*_*ρ snp*_ = 0.293 [0.237], e.g. highest repeatability site #32 Mallorca, Spain, *r*_*ρ snp*_ = 0.778 [95% CI: 0.762–0.794]). Repeatable trends across replicates can be further used to map genomic regions with more predictable frequency. Using a likelihood ratio test (LRT-1, Text S11) (23), we found that such signals were widespread along the genome, likely due to many alleles being linked to causal loci and thus experiencing indirect selection or “genetic draft” (24) (Fig. 2E). Overlaying the selection signals onto 16,917 linkage disequilibrium (LD) blocks along the *A. thaliana* genome estimated in the founder population (see Methods and Text S12) identified 377 LD blocks that showed repeatable signals within gardens and that overlapped in 10 or more gardens (overlap higher than expected by permutation test, *P* = 10^−6^, Fig. S31), suggesting that adaptation through standing variation may be rapid and highly polygenic.

An additional way to evaluate selection on standing variation is by analyses of accession sorting. We reconstructed the relative abundance of the 231 founder *A. thaliana* accessions over time using allele frequencies from pool-sequencing and the genomes of the founders. We note that although we detect some outcrossing (Text S10, Fig. S14), ignoring outcrossing allows us to implement this intuitive analysis of accession frequency evolution. Muller plots reveal patterns akin to strain evolution in microbial studies (Fig. 2B, Fig. S22, S23) where multiple adaptive variants are competing to rise in frequency (10). These dynamics are expected since the starting population was rich in standing genetic variation. Following the previous rationale that deterministic trends of accession relative frequency must be at least partially owed to differences in fitness of accessions, and thus natural selection, we also found high rank correlation of accessions relative frequency showcasing repeatable trends within garden environments (Fig. 2B,C, mean[sd] *r*_*ρ accession*_ = 0.194[0.179]). Another approach to the repeatability in frequency shifts used in evolve and resequence experiments has aimed to quantify the heritability of frequency changes (25). Having accession relative frequency in multiple replicates within a garden, heritability of frequency changes can be simply estimated using random effect regression (*H*^*2*^_*range*_ = 12.9–79.6%*, n*=30 gardens), and indeed strongly correlates with repeatability (correlation *H*^*2*^–repeatability, *r*=0.93, *P*=5.3×10^−14^, see Text S9).

Not only did similar accessions rise in frequency in replicates within an environmental garden, but they also prospered in parallel across gardens of similar climates. For example, we see strongly parallel changes in three cold locations in Germany (mean *r*_cold_= 0.451 [95% CI= 0.459–0.443]), and three warm locations in south Spain (*r*_warm_ = 0.453 [95% CI=0.437–0.468], Fig. S29), indicating similar relative fitness ranks of accessions are maintained in similar environments even in geographically distinct locations (26, 27). Correlations among replicates within gardens were naturally higher than correlations between gardens of similar climates (*r*_*cold within*_ = 0.548 [0.534–0.562] *, r*_*warm within*_ = 0.699 [0.682–0.716], Fig. S29), which may be attributed to technical factors in the experimental design (e.g. correlated experimenter temporal sampling, or dispersal among trays, although our dispersal estimates indicate <1% of seeds in a tray could be migrants, Fig. S3). Alternatively, these results may indicate that the environmental selection pressures are complex and unique within each garden, even if we classify several gardens as belonging to similar climates.

We conclude that patterns from genomic time series support non-neutral, natural-selection-driven evolutionary dynamics, presumably involved in rapid adaptation. Under such rapid adaptation we may expect populations that are initially maladapted would decline and then rebound as adaptive genotypes rise in frequency (28). By tracking population sizes through annual census (Text S1), eight out of 30 experimental gardens showed average significant signs of population recovery across replicates in the third generation, with U-shaped trajectories reminiscent of evolutionary rescue (Fig. 2F, Fig. S7, S8, S20, Text S8). Together, the significant allele frequency shifts and the U-shape population size trajectories support the notion that adaptive evolutionary rescue occurred across climates.

## Rapid evolution follows the pattern of past local adaptation

**Fig. 3.**
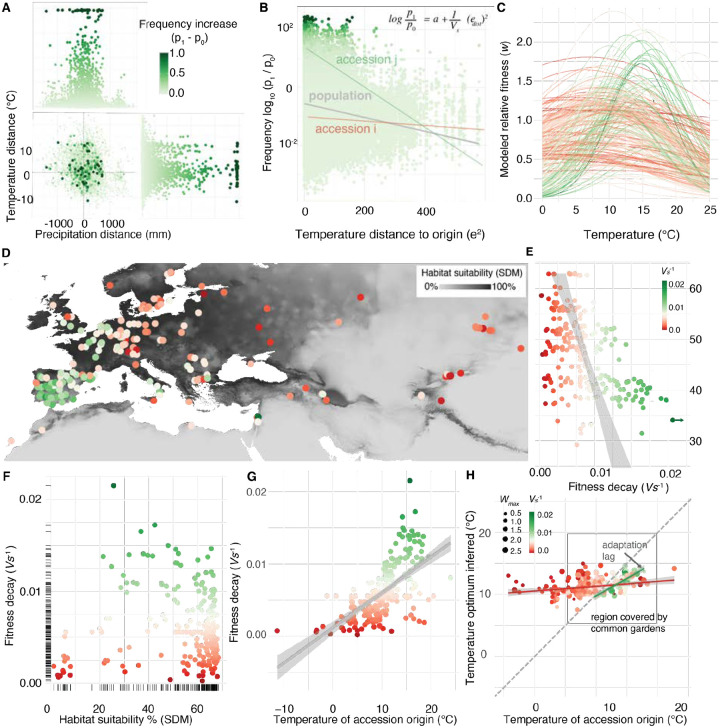
Rapid evolution follows local adaptation. (**A**) Accession relative frequency change (*p*_*1*_−*p*_*0*_) over climatic distance across all three years (*n* = 75,075 garden-accession origin transplant combinations) showing that planted accessions at sites with annual temperature and precipitation most similar to their home environment typically increase in frequency more than those transplanted to climatically distant environments. (**B**) Transformation of data in (A) to display *log (p*_*1*_*/p*_*0*_*)* and squared temperature distances to fit a model of stabilizing local adaptation. Grey line regression represents the average fitness decline of the GrENE-net accessions with climate distance transplant (i.e. the stabilizing selection parameter *V*_*s*_^*211*^) while accessions *i* and *j* are examples of accession-specific *V*_*s*_^−*1*^ slopes. (**C**) Idealized stabilizing selection curves for all 231 accessions based on fitting (B) equation of *V*_*s*_ *and W*_*max*_. (**D**) Per-accession local adaptation parameter *V*_*s*_^−*1*^ visualized in a map of the accessions’ geographic collection of origin colored by habitat suitability and (**E**) across a latitudinal gradient of accession’s location origin. (**F**) Relationship between the strength of the per-accession local adaptation parameter and habitat suitability of the accessions’ locations of origin and (**G**) the accessions’ temperatures of origin. (**H**) Annual temperature averages at accessions’ origins against temperatures of gardens weighted by the accessions’ frequency, as a proxy of temperature optimum. The gray lines represent regression lines, and the shaded areas indicate their confidence intervals.

The strong evidence of rapid adaptation in our experiment is likely attributable to the fact that we drew lines from natural populations, which presumably were locally adapted to their different native conditions. We next wanted to determine if our observed rapid adaptation mimics past local adaptation to climatic conditions. Previous studies have found local adaptation in *A. thaliana* (26, 27, 29–32) and many other species (15, 16). Here, we used information on each accession’s climate of origin (Fig. 1A) and change in frequency in the experiment (Fig. 3A) to determine if genotypes from matching climatic origins increased in frequency. We focused on the first generation sequencing since sampling reproductive adults is a proxy of relative fitness per accession (since *p*_*t+1*_ */ p*_*t*_ = *w / ŵ*). We indeed found a strong negative correlation between accession’s relative frequency in one generation and increasing climate distance squared across all gardens (*r*_*ρ*_ = −0.25, *P*< 2×10^−16^ , *n* = 169,115) (Fig. 3A, Fig. S32, S34, S35, S42, see Text S13). To formally quantify climate-driven natural selection, we used a Gaussian stabilizing natural selection model (17) extended to accession frequency measurements in experimental evolution: *log(p*_*t+1*_ */ p*_*t*_*)* = *log(W*_*max*_ */ ŵ) - V*_*s*_^−*1*^ *(z*_*origin*_ *- z*_*garden*_*)*^*2*^. Here, *W*_*max*_ denotes the accession-specific maximum fitness at the origin environment; *V*_*s*_^−*1*^ denotes the accession-specific strength of natural selection measuring the rate of fitness decay; *ŵ* denotes garden-specific average fitness; *z*_*garden*_ denotes the garden environment and *z*_*origin*_ denotes the accession-specific optimal environment (assumed to be the climate of accession origin described by a chosen environmental variable. See details in Methods). With this framework, we quantified the strength of climatic local adaptation for each of 19 temperature and precipitation climate variables (BIOCLIM variables calculated from ERA5-land database, Fig. S34, S40, (33)). We found evidence of climate adaptation from both temperature and precipitation variables, with the strongest local adaptation signal being annual mean temperature (BIO1) (*R*^*2*^ = 0.337, *P* < 2.2×10^−16^, *n* = 71,976, Fig. 3A, B, Fig. S33).

To understand possible differences between “accession niches”, we expanded the Gaussian framework to be accession-specific (Fig. 3B, C, Fig. S39). This revealed a trade-off between maximum fitness and rate of fitness decay across environments (Fig. 3H, Fig. S38) (17), whereby accessions’ maximum fitness correlated with more rapid fitness decay when planted in a garden with a different temperature profile (*r*_*ρ Wmax* and *Vs-1*_ = 0.344, *P* = 8.086×10^−8^, *n* = 231, Fig. 3B, Fig. S41). These results are reminiscent of the ecological trade-off observed between specialists and generalists (27, 34). Consequently, we found that “generalist” accessions with wider niches (low *V*_*s*_^*−1*^) are originally from regions of lower habitat suitability at the species distribution range, and typically from colder regions (Fig. 3E, F, Fig. S10 see Methods) (17), while “specialist” accessions with narrower but higher fitness curves appear to come from central-to-warmer native environments (*r*_*Vs* − *temp*_ = 0.634, *P*= 2.2×10^−16^, *n*=231, Fig. 3G) (35). For those accessions with narrower niches (*1/V*_*s*_ *>0.15*), where the local adaptation signal is strongest, we found a notable adaptation lag (17, 36), whereby accession’s realized optimum estimated from the experimental gardens was on average colder than the current climate at their geographic origin, on par with the magnitude of ~1.5°C climate change to date (37) (Estimated optimum - origin temperature = −1.87°C [IQR = −0.796 – −2.84°C], Fig. 3H).

Because natural selection ultimately operates on phenotypes, we sought to identify the phenotypic basis of rapid adaptation across gardens. Our field observations revealed that spring flowering rapidly synchronized with the expected growth season along a latitudinal temperature gradient within three years (*r*_*y1*_ = 0.347, *P*= 2.89×^−31^*, n*=1060*; r*_*y2*_ = 0.526, *P*= 9.14×^−60^*, n*=822*; r*_*y2*_ = 0.554*, n *= 535, *P*= 2.67×^−44^, Fig. S6). Specifically, flowering periods extended to July in high latitudes and started as early as February in low latitudes (Fig. S4). To extend our phenotypic evolution study to phenotypes that are difficult to measure in the field, we used a curated and imputed database of traits of known heritability across *A. thaliana* accessions (38) with GrENE-net founder accessions (*n*=213). By correlating *ex situ* phenotype with the accession’s relative frequency change at each garden (Text S15), we found evidence that a number of traits likely diverged across environments (Fig. S44). For instance, as proof of concept, using the highly heritable flowering time measured in growth chambers (*h*^*2*^_*kinship*_ = 0.93 [95%CI 0.898–0.975]), we found accessions with known late flowering times showed a weak but significant correlation in the coldest locations (*r*_*freq* − *ft*._ = 0.074, *P* = 4.6×10 ^−6^, site #27 Tartu, Estonia, ~5°C mean annual temperature), while in warm locations the correlation was strong and reversed, with early-flowering accessions becoming more common (*r*_*freq - ft*._ = −0.25, *P* = 1.1×10^−117^, site #5 Madrid, Spain, ~14°C mean annual temperature). Similarly, strong seed dormancy of accessions (i.e., days of seed dry storage required to reach 50% germination, DSDS50, *h*^*2*^_*kinship*_ = 0.987 [95%CI 0.934–0.998]) correlated with accession relative frequency increase in warm, low precipitation environments (<100 mm summer rain [BIO18], Madrid (Spain), and Lesbos (Greece), *r *= 0.24–0.25, *P* < 10^−104^), where strong dormancy prevents summer germination and increases bet-hedging (39–41). This follows an expected growth season gradient from late flowering and high autumn germination in high latitudes to early flowering and high seed dormancy in low latitudes (41, 42). Beyond phenology, we found a suite of other traits associated with climatic evolution, such as increased leaf area in cold environments (e.g. <10°C, Warsaw, Poland, *r*_*leaf area*_ = 0.115, *P* = 2.09×10^−7^), or decreased leaf stomatal density in summer dry environment (<20 mm summer precipitation, Lesbos, Grece, *r*_*stomata*_ = −0.07, *P* = 1.8×10^−5^) (see different phenotypes and environments: Fig. S43 S44, Table S7). Together, this supports the hypothesis that rapid adaptation trends across local environments are also driven by phenotypic evolutionary divergence.

## Mapping the genetic basis of climate adaptation

**Fig. 4.**
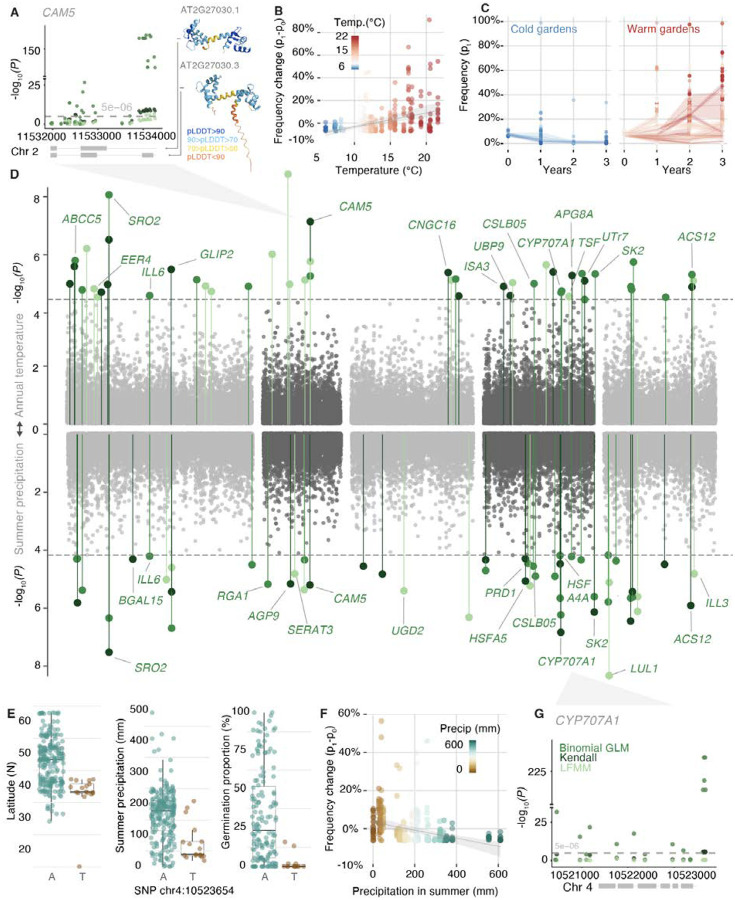
Rapid adaptation signals along the *A. thaliana* genome Experimental-evolution Genome-Environment Associations (eGEA) of rapid allele frequency trajectories with temperature (**A-D**) and precipitation in summer (**D-G**) using three statistical approaches: Latent Factor Mixed Model (LFMM), quasi-binomial Generalized Linear Mixed model (GLM), and Kendall correlation. (**A**) Zoom into the temperature Manhattan plot with *CAM5* SNP associations, reporting *P-values* obtained from the three models before inflation correction with WZA. The protein structures (AlphaFold computed) of two alternative splicing isoforms of the *CAM5* gene are depicted: isoform 1 (AT2G27030.1) and isoform 3 (AT2G27030.3). Grey boxes along the genome (x-axis) indicate two gene models of the TAIR reference genome which are present in published transcriptome data (54). (**B**) Example of divergent allele frequency trajectories of the *CAM5* top allele (chr4:11533937) across experimental locations along a temperature gradient. (**C**) Frequency trajectories of top *CAM5* allele over years separating experimental gardens in high (>10°C) and low (<10°C) mean annual temperature. (**D**) Manhattan plot of eGEA association of mean annual temperature (up) and summer precipitation (down) combining results from the three applied statistical approaches with haplotype block *P-value* pooling with WZA. Five *A. thaliana* chromosomes indicated in grey and black. (**E**) Relation of the top *CYP707A1* SNP allele identified in precipitation eGEA and boxplots of allele distribution relative to accession origin latitude (left) and precipitation (mid), and the expected effect of reduced germination (right). (**F**) Relation between changes in *CYP707A1* alleles and precipitation in summer. (**G**) CYP707A1 gene model and zoom into top SNP associations, reporting *P-values* obtained from the three models before inflation correction with WZA, the grey boxes along the genome (x-axis) indicate the gene model of the TAIR reference genome.

To map the genetic basis of climate adaptation, we scanned the genome for highly divergent allele frequencies across gardens. We used experimental evolution Genome-Environment Associations (eGEA) to identify SNP frequency changes across experimental gardens associated with environmental selective forces as reflected in BIOCLIM variables (see Methods). We used three modeling frameworks: a Latent Factor Mixed Model (LFMM) to account for population structure (43), a binomial GLM to account for variable population sizes, and Kendall-τ ranked correlations (see Methods) to detect nonlinear associations in combination with an LD block partition and *P*-value pooling with WZA (Fig. S46, S47, S48) (44). After false discovery rate (FDR) correction, we identified 44 significant blocks associated with multiple climate variables (Fig. 4A Dataset S9). Several blocks included genes known to affect growth, flowering and dormancy while other blocks included genes that are likely involved in environmental stress responses (Text S17, Table S9, S10). We then compared our experimental evolution eGEA with classic population GEA (or climate GWA). The classic GEA approach uses genomic sequencing of natural populations and directly associates genetic variants with the climate of collection of accessions to find enrichments, which requires careful population structure correction as population history correlates both with geographic and genetic patterns. This classic GEA approach has been used previously to map climate adaptation in *A. thaliana* populations (30). Instead, our eGEA uses the fact that standing genetic variants start at equal frequency across all experiments, and climate-driven natural selection will increase or decrease their frequencies over time. We surprisingly found little overlap in the top significant genomic LD blocks (0–3 overlapping FDR significant blocks across 19 BIOCLIM variables (Fig. S49, Table S16. See classic GEA in Text S16 and interpretations of partial overlap). Regardless, this novel experimental evolution eGEA confirmed well-known loci or revealed novel genes important for climate adaptation.

A gene well-known to be involved in spring flowering we identified in our eGEAs is the “florigen”-encoding gene *TWIN SISTER OF FT* (*TSF,* AT4G20370) (45–47) (LFMM-WZA block significance *P* = 3.9×10^−5^; LFMM of lead SNP, *P* = 3.63×10^−7^, Kendall *P* = 7.2×10^−10^, binomial GLM *P* = 3.55×10^−42^) (Fig. S55, S56, Text S17). We observed SNPs significantly shifting frequency across the experimental temperature gradient in the same genomic region detected in an earlier local adaptation study (48). *TSF* alleles were previously associated with flowering time variation within the Iberian Peninsula (Spain and Portugal) both in natural populations and common gardens (49, 50). In our study, we also found that the accessions carrying the top *TSF* alleles had a significantly earlier flowering times (Wilcoxon tests, *P* < 0.05, *n* = 220, Fig. S57, Table S18).

For an annual plant, both onset of flowering and the timing of germination determine adaptation to seasonal climates, and vary strongly across the *A. thaliana* range (41). It is thus no surprise that we also found strong genotype-environment associations for dormancy-related genes such as *CYTOCHROME P450 (CYP707A1)* (Fig. 4D–F), a gene encoding an ABA-catabolic enzyme highly expressed during germination (51, 52). This gene acts to promote germination by reducing ABA accumulation (52). The alternate *CYP707A1* allele became enriched in dry study sites (<80 mm summer precipitation) (Fig. 4F–G). In nature, this allele is mainly detected at lower latitudes with extremely low germination rates in the laboratory (mean germination % difference = −30%, Wilcoxon test, *n*=220, *P* = 2.648×10^−6^, Fig. 4E). This suggests that there has been rapid adaptation through changes in seed dormancy timing in our experimental evolution plots.

Our eGEA also identified genes that have not been previously implicated in climate adaptation. We identified a strong significant association with variation in *CALMODULIN 5* (*CAM5*) in all three eGEA methods (LFMM-WZA *P* = 2×10^−6^, Kendall’s τ WZA *P* = 1×10^−7^, binomial GLM WZA *P* = 8×10^−6^, Fig. 4B) (see Text S17). Calmodulins bind stress-triggered calcium to modulate signaling in the context of environmental stress or pathogen responses. *CAM5* expression has been shown to be triggered by high temperature exposure in laboratory conditions (53). We found that the top associated SNPs are located in the intron before a third exon that is alternative spliced (Fig. 4A). Accessions from warm environments tend to have increased expression of a *CAM5* isoform that includes the alternative third exon downstream of the intron with the top SNP (54) (*r *= 0.124, *P* = 0.004*, n *= 521, Fig. S53). In concordance with this prediction, we find that frequencies of alternative alleles in the second *CAM5* intron increase in frequency over time in warm gardens (change rate: +1%/year, *P* = 3.58×10^−15^), and decrease in cold gardens (change rate: −1.6%/year, *P* = 1.6×10^−18^, Fig. 4B, C). Taking gardens in both extremes of the temperature gradient, either cold or warm, we estimated selection coefficients on *CAM5* in Cadiz (Spain) to be *s *= 57% (95% CI *s* = 49% – 66%, *p*_*year3*_ = +46%) and in Brixen im Thale (Austria), *s* = −47% (95% CI = −56 – −38%, *p*_*year2*_ = 0.2%, Fig S52, see selection estimation in Text S18). Other eGEA hits with links to stress responses include genes encoding heat shock transcription factors *(HSF4A4, HSFA5)* or an aquaporin-like protein (Fig. 4D, Text S17). The magnitude of environment-driven natural selection we inferred on *CAM5* was highly significant but hardly unique, with abundant polygenic signals detected along the genome (Fig. 4D). These findings are on par with our observed genome-wide patterns of large selection coefficients and rapid evolutionary responses, akin to those seen in fruit flies or stick insects adapting to seasonal environments (4, 55).

## The direction of rapid evolution across climates is predictable

**Fig. 5.**
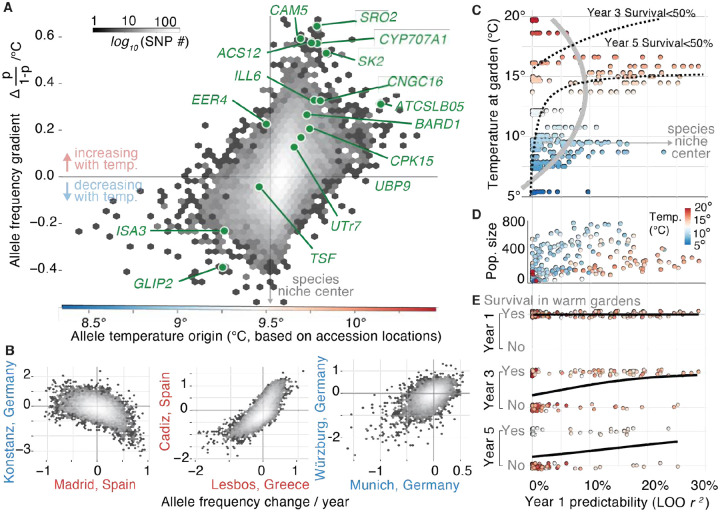
Predictability of genome-wide evolution and population survival across environments (**A**) Allele frequency changes with temperature (logistic parameter *β* = Δ*p/(1-p)*/°C) and its relation to allele’s temperature origin based on the average annual temperature of the *A. thaliana* accessions carrying such alleles. Logistic regression *β* was calculated per allele and averaged within each LD block (n = 16,656). Top gene associations (Fig. 4) are highlighted in green. (**B**) Example of allele frequency trajectory over time fitting a logistic regression (Δ*p/(1-p)*/year), comparing several warm (>10 °C, red) and cold (<10°C, blue) experimental gardens (Fig. S69). (**C**) Leave-one-out (LOO) predictability of year 1 evolutionary trends (*log(p*_*1*_*/p*_*0*_*)*) per replicate (*n* = 325) based on new genomic offset and stabilizing selection across gardens of different temperatures (see other metrics Fig. S69). Grey line indicates the fitted second term polynomial between predictability and temperature. Dotted lines indicate isolines of population survival from fitted logistic regressions in (E). Species niche center represents the average temperature of origin across all founder accessions (9.6°C). (**D**) Relationships between LOO predictability (year 1) and population size over time (summed total number of individuals sampled year 1–3). (**E**) Logistic regressions of LOO predictability of evolutionary trends of population replicates and survival in the 1^st^, 3^rd^, and 5^th^ years.

There is an urgent need to predict potential (mal)adaptation of species to future climates, both for species of conservation focus as well as domesticated species (56). We thus asked whether the observed changes in allele frequency (*p*_*0*_*→p*_*1*_) across experimental evolution gardens could have been predicted from knowledge of the genetic basis of local adaptation of the species. We reason that the climatic factors that drive differences in survival in experimental gardens likely also occur in natural populations, so we can use local adaptation signals of natural populations as a predictive signal (57). In agreement with this rationale, we found that alleles of warm origins showed upward frequency trajectories (Δ *p/(1-p)*/°C) in warm experimental gardens and downward trajectories in cold sites (Fig. 5A, *R*^*2*^ = 0.242, *P* < 2.2×10^−100^, see Methods, Fig. S45). Likewise, experimental gardens in similar climates showed concordant changes in allele frequencies (e.g. Madrid vs Barcelona [Spain], *r* = 0.657, *P* < 2.2×10^−100^, and Cádiz [Spain] vs Lesbos [Greece] *r* = 0.733, *P* < 2.2×10^−100^, Fig. 5B), whereas gardens of contrasting climates showed opposite trajectories (Fig. 5B Konstanz [Germany] vs Madrid [Spain], *r* = −0.393, *P* < 2.2×10^−100^, and Warsaw [Poland] vs Madrid [Spain], *r* = −0.262 *P* = 3.8×10^−165^, *n* = 16,757 LD blocks) (see systematic analyses of antagonistic pleiotropy in Text S19 and Table S11). Such a strong signal is likely driven by a combination of high polygenicity of adaptation, antagonistic pleiotropy, and genome-wide linkage disequilibrium (27, 58). Using this signal, we fitted so-called “genomic offset” models that assign genotypes’ or populations’ fitness scores based on the allelic associations with climate (59–61) (GO_score_ = *(1/n)* Σ *(|p*_*adapt.,i*_ − *X*_*accession,i*_*|),* see Methods). Using leave-one-out (LOO) cross-validation, we aimed to predict evolutionary trajectories for each garden (*p*_*0*_*→p*_*1*_) using the other gardens as genomic offset training data (Fig. 5C, Fig. S62). This showed substantial rank predictive accuracy across all gardens (*r*_*ρ*_ = 0.263 [IQR 0.160–0.368], *P* = 5.752×10^−7^, *n* = 325; *r*^*2*^ range = 0–10.9%) beyond what climate distance alone could predict (*r*_*ρ*_ = 0.181 [IQR 0.020–0.332], *n* = 325; *r*^*2*^ range = 0–10.1%), which is in agreement with previous findings in common gardens of *A. thaliana*, steppe grasses, and poplar tree provenances (59, 62). A similar genomic offset model that additionally incorporated the Gaussian stabilizing local adaptation parameters, *V*_*s*_^−*1*^ *W*_*max*_, has a similar or higher cross-validation predictability (*r*_*ρ*_ = 0.415 [IQR 0.311–0.544], *P* < 2.2×10^−16^, *n* = 325, *r*^*2*^ range = 0–29.3%, Fig. S63, S64, see Methods).

We ultimately predict early signs of rapid evolution to be informative about long-term population survival in changing climates. So far, evolutionary studies in the wild have typically been limited by either the breadth of climate gradients studied or by the studies’ short duration (63). To address this gap, we leveraged the geographic span of our experimental evolution plots and the census monitoring for up to five years. First, we tested whether predictability of early rapid evolution trends from genomic offset varies across climates. Correlating environmental data with predictability metrics, we found evolutionary predictability increased with annual temperature (14°C–17°C) but had a significant concave drop at high (>18°C) annual temperatures (regression’s quadratic coefficient = −0.001, *P* = 2.24×10^−9^, Fig. 5D, Fig. 2C). In contrast, predictability remained lower in cold and wet environments, where mortality was rare and natural selection was presumably low (Fig. 5C). Second, we used logistic regression-based methods to test whether evolutionary predictability was associated with survival or extinction of experimental population replicates. We found climate, evolutionary predictability, as well as their interaction to be generally significant (5E; logistic regression 3^nd^ year survival: *P*_*pred*._ = 0.02, *P*_*temp*._ = 0.02, interaction *P*_*pred*. × *temp*_ = 0.03; 5^th^ year survival: *P*_*pred*._ = 0.01, *P*_*temp*._ = 0.02, *P*_*pred*. × *temp*_ = 0.02; Fig. S70). The significant interaction indicated that evolutionary predictability correlated with increased survival especially in the warmest climates: for instance, the likelihood of population survival at ~15°C annual temperature is over 50% only when initial evolutionary predictability is *r*^*2*^>15% (see isolines, Fig. 5C). This reminds us of eco-evolutionary tipping points that have been long theorized in population genetic literature (64), where in extreme environments natural selection increases mortality and overpowers the efficiency of evolutionary adaptation, leading to erratic evolutionary trends. Given natural populations of short-lived plants and animals show evidence of evolutionary and demographic responses in sub-decadal scales (4, 55, 65, 66), our results should be helpful to downscale predictions to conditions with limited genetic diversity or less extreme climate gradients. In the future it will be key to better understand eco-evolutionary tipping points across species that may help us anticipate when species’ evolutionary responses may succeed or fail under climate change (67).

## Supplementary Material

1

## Data Availability

Supplemental Tables and Datasets are available in the online version of the paper and in www.GrENE-net.org/data and Github: https://github.com/moiexpositoalonsolab/grene. Founder genomes are available at http://1001genomes.org/data/GMI-MPI/releases/v3.1/. Sequencing Illumina reads for GrENE-net experimental evolution plots are deposited at NCBI with accession number https://www.ncbi.nlm.nih.gov/sra/PRJNA1256468. Processed frequency files are available at www.GrENE-net.org/data. Scripts to reproduce analyses and figures are available at: https://github.com/moiexpositoalonsolab/grenephase1-paper. Both intermediate data and scripts are available also at Zenodo with doi: … Software to analyze Pool-seq data: g_r_enepipe and g_r_enedalf are available on Github: github.com/moiexpositoalonsolab/grenepipe, github.com/lczech/grenedalf, github.com/moiexpositoalonsolab/hapfire. The 1001G seed collection can be obtained from the Arabidopsis Biological Resource Center (ABRC) under accession CS78942.
